# Revolutionizing Cancer Treatment: Unveiling New Frontiers by Targeting the (Un)Usual Suspects

**DOI:** 10.3390/cancers16010132

**Published:** 2023-12-27

**Authors:** Valerio Costa, Elisa Giovannetti, Enza Lonardo

**Affiliations:** 1 Institute of Genetics and Biophysics (IGB), National Research Council of Italy (CNR), 80131 Naples, Italy; valerio.costa@igb.cnr.it; 2 Department of Medical Oncology, Amsterdam UMC, VU University, Cancer Center Amsterdam, 1081 HV Amsterdam, The Netherlands; e.giovannetti@amsterdamumc.nl; 3 Fondazione Pisana per la Scienza, San Giuliano Terme, 56124 Pisa, Italy

This Special Issue includes original articles and reviews on both established and innovative approaches to cancer targeting, showcased at the 29th IGB Workshop titled “Targeting the (un)usual suspects in cancer” “https://29thigbworkshop.sciforum.net/ (accessed on 19 December 2023), held on 2–3 December 2021.

Since the inception of the “war on cancer” 52 years ago through the US National Cancer Act, numerous therapeutic strategies promoting the death of cancer cells have been developed, resulting in a diverse range of available cancer treatments [1,2]. However, conventional treatments often lack selectivity in killing tumor cells, facing challenges due to the tumor heterogeneity [3,4]. In this context, the identification of new drugs or drug combinations targeting well-known signaling pathways [5,6], along with the repurposing of approved drugs with undiscovered antitumor activities [7,8,9], offer refreshed arrays of therapeutic options in oncology. Among them, anastrozole, an aromatase inhibitor, demonstrated new antitumor activities in breast cancer [10].

Federico et al. introduced an innovative network pharmacology strategy, combining mechanistic and chemocentric approaches to drug repositioning [11]. This involves a multilayer network-based computational framework integrating disease perturbational signatures with drug intrinsic characteristics, encompassing factors such as their mechanism of action and chemical structure. Public data from The Cancer Genome Atlas were used [https://www.cancer.gov/tcga (accessed on 27 November 2023)], identifying paclitaxel as a promising candidate for combination therapy across various cancer types [11]. Furthermore, in the spirit of drug repositioning, the analysis identified several non-cancer-related unconventional drug targets as potential candidates for combinatorial pharmacological intervention in cancer treatment. These include hormonal drugs (carbimazole and methimazole), psychoanaleptics (phenelzine, tranylcypromine, and pentobarbital), calcium channel blockers (perhexiline), and antihypertensives (clonidine). These promising findings support the use of this framework as a tool to facilitate the prioritization of drug combinations and repositioning by integrating the mechanistic characteristics of the disease with the intrinsic properties of the drugs.

A different approach was described by Casalino et al., who reviewed diverse strategies aiming to inhibit tumor growth, metastasis, and drug resistance by targeting the FOS-family transcription factor Fra-1, encoded by *FOSL1* gene, which has emerged as a notable therapeutic target within the AP-1 complex [12]. Fra-1 is frequently overexpressed in various solid tumors, triggered by major oncogenic pathways like BRAF-MAPK, Wnt-beta-catenin, Hippo-YAP, and IL-6-Stat3. In agreement with new approaches targeting transcription factors in cancer [13], the discussed strategies include the design—and tumor-specific delivery—of Fra-1/AP-1-specific drugs, RNA-based therapeutics targeting the *FOSL1* gene, its mRNA, or regulatory circular RNAs (circRNAs), blocking peptides, small-molecule inhibitors, and innovative Fra-1 protein degraders.

A novel therapeutic strategy was also described in the study by Che et al. [14], using a previously established array of pancreatic ductal adenocarcinoma (PDAC) cell lines, primary cultures, and chorioallantoic membrane models [15]. This strategy involved the utilization of nanotechnology for the delivery of chemotherapeutics, with a preference for radiosensitizing agents, aiming to enhance the efficacy of chemoradiation. The study specifically assessed the impact of biodegradable ultrasmall-in-nano architectures (NAs) containing gold ultra-small nanoparticles enclosed in silica shells loaded with a cisplatin prodrug (NAs-cisPt) in combination with ionizing radiation (IR). The main findings highlighted the heightened cytotoxic effect of NAs-cisPt, particularly through the controlled release of the cisplatin prodrug [14]. Given cisplatin’s recognized role as a radiosensitizer [16], the administration of the cisplatin prodrug in a controlled manner through encapsulation presents a promising and innovative treatment approach. Moreover, a recent study showed that PDAC paclitaxel-resistant cells exhibit enhanced sensitivity to IR due to the greater accumulation of DNA damage depending on the radiation-induced modulation of autophagy and of the Hippo pathway [17], prompting further research on new strategies to promote the antitumor effects of IR in PDAC [18].

Apart from key genetic factors, desmoplasia and the tumor microenvironment (TME) have been recognized as key contributors to PDAC chemoresistance [19,20], and Gregori et al. integrated biomechanical and pharmacological approaches to investigate the role of the cell-adhesion molecule Integrin Subunit Alpha 2 (ITGA2), a crucial regulator of the extracellular matrix [21], in PDAC resistance to gemcitabine [22]. Notably, high ITGA2 expression was correlated with shorter progression-free and overall survival, indicating its prognostic significance and association with gemcitabine treatment ineffectiveness. Transcriptomic and proteomic analyses revealed upregulated ITGA2 in gemcitabine-resistant cells, whereas silencing ITGA2 reduced the aggressive behavior of these cells both in vitro and in vivo, associated with the upregulation of phospho-AKT.

Notably, the PI3K/AKT/mTOR signaling pathway, a crucial downstream effector of KRAS, plays a significant role in regulating key hallmarks of cancer [23], and several studies support the application of agents targeting the PI3K/AKT/mTOR pathway in the context of PDAC [24,25,26]. However, for certain tumors, such as colorectal and PDAC, even highly selective therapies fail to completely eradicate the disease because they do not target the niche of cancer stem cells (CSCs), capable of reconstituting and perpetuating malignancy [27]. Therefore, targeting pathways specific to the maintenance of CSCs and disrupting communication between tumor cells and the TME are emerging as additional fundamental approaches in the ongoing “war on cancer” [28,29].

For instance, a recent study reported a LAMC2-expressing cell population, which is endowed with enhanced self-renewal capacity and is sufficient for tumor initiation and differentiation and driving metastasis [30]. The profiling of these cells indicated a prominent squamous signature and differentially activated pathways critical for tumor growth and metastasis, including the deregulation of the TGF-β signaling pathway [30], a key pathway in the biology of cancer progression [31]. Treatment with Vactosertib, a new small-molecule inhibitor of the TGF-β type I receptor, completely abrogated lung metastasis, primarily originating from LAMC2-expressing cells [30].

Lastly, the list of tumor hallmarks and cancer-causing factors has been updated in the last decade to include new cellular processes (e.g., metabolism; [32,33]) and molecular factors (e.g., non-coding RNAs; [34]) as significant contributors to tumor onset, progression, and drug sensitivity. Despite metabolic alterations being reported approximately a century ago [35], targeting tumor metabolism has recently regained interest as a plausible interventional strategy [36,37]. Additionally, mounting evidence suggests a previously unrecognized role for long non-coding RNAs (lncRNAs) as oncogenes and tumor suppressors due to their ability to regulate various cancer hallmarks. These findings, particularly the cancer-specific expression of most lncRNAs, establish the rationale for considering lncRNAs as therapeutic targets [38,39], as silencing them would not induce side effects in other tissues or cell types.

In summary, echoing the famous sentence expressed in the renowned crime movie *The Usual Suspects*, which states, “*The greatest trick the devil ever pulled was convincing the world he didn’t exist*”, it becomes apparent that concentrating solely on recognized oncogenic factors can narrow our perspectives and limit the effectiveness of existing cancer therapies [40]. This underscores the need to delve into novel therapeutic targets and approaches, encompassing new strategies that involve the individual—or in some cases simultaneous—targeting of the TME, metabolism, CSCs, and non-coding RNAs.
